# Plasma and memory B cell responses targeting O-specific polysaccharide (OSP) are associated with protection against *Vibrio cholerae* O1 infection among household contacts of cholera patients in Bangladesh

**DOI:** 10.1371/journal.pntd.0006399

**Published:** 2018-04-23

**Authors:** Amena Aktar, M. Arifur Rahman, Sadia Afrin, Aklima Akter, Taher Uddin, Tahirah Yasmin, Md. Israk Nur Sami, Pinki Dash, Sultana Rownok Jahan, Fahima Chowdhury, Ashraful I. Khan, Regina C. LaRocque, Richelle C. Charles, Taufiqur Rahman Bhuiyan, Anjali Mandlik, Meagan Kelly, Pavol Kováč, Peng Xu, Stephen B. Calderwood, Jason B. Harris, Firdausi Qadri, Edward T. Ryan

**Affiliations:** 1 International Centre for Diarrhoeal Disease Research, Dhaka, Bangladesh; 2 Division of Infectious Diseases, Massachusetts General Hospital, Boston, Massachusetts, United States of America; 3 Department of Medicine, Harvard Medical School, Boston, Massachusetts, United States of America; 4 NIDDK, LBC, National Institutes of Health, Bethesda, Maryland, United States of America; 5 Department of Microbiology and Immunobiology, Harvard Medical School, Boston, Massachusetts, United States of America; 6 Division of Global Health, Massachusetts General Hospital for Children, Boston, Massachusetts, United States of America; 7 Department of Pediatrics, Harvard Medical School, Boston, Massachusetts, United States of America; 8 Department of Immunology and Infectious Diseases, Harvard T. H. Chan School of Public Health, Boston, Massachusetts, United States of America; University of Tennessee, UNITED STATES

## Abstract

**Background:**

The mediators of protection against cholera, a severe dehydrating illness of humans caused by *Vibrio cholerae*, are unknown. We have previously shown that plasma IgA as well as memory B IgG cells targeting lipopolysaccharide (LPS) of *Vibrio cholerae* O1 correlate with protection against *V*. *cholerae* O1 infection among household contacts of cholera patients. Protection against cholera is serogroup specific, and serogroup specificity is defined by the O-specific polysaccharide (OSP) component of LPS. Therefore, we prospectively followed household contacts of cholera patients to determine whether OSP-specific immune responses present at the time of enrollment are associated with protection against *V*. *cholerae* infection.

**Methodology:**

In this study, we enrolled two hundred forty two household contacts of one hundred fifty index patients who were infected with *Vibrio cholerae*. We determined OSP-specific memory B cells and plasma IgA, IgG and IgM antibody responses on study entry (day 2).

**Principle findings:**

The presence of OSP-specific plasma IgA, IgM, and IgG antibody responses on study entry were associated with a decrease in the risk of infection in household contacts (IgA, p = 0.015; IgM, p = 0.01, and IgG, p = 0.024). In addition, the presence of OSP-specific IgG memory B cell responses in peripheral blood on study entry was also associated with a decreased risk of infection (44% reduction; 95% CI: 31.1 to 99.8) in contacts. No protection was associated with cholera toxin B subunit (CtxB)-specific memory B cell responses.

**Conclusion:**

These results suggest that immune responses that target OSP, both in plasma and memory responses, may be important in mediating protection against infection with *V*. *cholerae* O1.

## Author summary

*Vibrio cholerae* is a non-invasive pathogen which causes watery diarrheal diseases both in adults and children. Natural infection with *Vibrio cholerae* provides protection against subsequent diseases and protection against cholera is serogroup specific. Serogroup specificity is defined by O-specific polysaccharide (OSP) of *V*. *cholerae*. In this study, we have found that uninfected household contacts had higher baseline OSP-specific plasma IgA, IgG and IgM antibody responses than infected contacts. These observations demonstrate those plasma antibodies responses against OSP are associated with a decrease of the risk of infection of household contacts of cholera patients. We also found that OSP-specific IgG memory B cells are associated with a decrease in the risk of infection in contacts of cholera patients. This result further supports the hypothesis that immune responses targeting *V*. *cholerae* OSP is a prime mediator of protection against cholera, and suggests that future work should focus on more detailed analysis of mucosal immune responses targeting OSP, as well as evaluation of potential mechanisms of how antibodies targeting *V*. *cholerae* OSP might mediate protection against cholera.

## Introduction

Cholera is a severe acute watery diarrhea of humans caused by *Vibrio cholerae* [1]. More than 200 serogroups of *Vibrio cholerae* have been identified, with serogroups O1 and O139 being associated with epidemic cholera. The mediators of protection against cholera are currently unclear. A growing body of evidence suggests that immune responses that target *V*. *cholerae* O-specific polysaccharide (OSP) may be a central mediator of such protection [2–8]. Protection against cholera following wild-type disease is relatively long-lived, lasting at least 3 to 10 years [9–11]. We have recently shown that patients recovering from cholera develop prominent plasma and memory B cell responses targeting *V*. *cholerae* OSP [2,4,8]. Whether such responses are associated with protection against cholera is uncertain and was the focus of this current study. We have previously found that approximately 25–30% of household contacts of cholera index patients have evidence of *V*. *cholerae* infection within 9 days of follow-up [6,7]. In this current analysis, we focused on whether baseline plasma and memory B cell responses against *V*. *cholerae* OSP in household contacts correlated with the risk of *V*. *cholerae* in the next 9 days.

## Methods and materials

### Study design and enrollment of participants

Patients hospitalized with cholera at the International Centre for Diarrhoeal Disease Research, Bangladesh (icddr,b) hospital in Dhaka, Bangladesh, and their household contacts, were enrolled in the study following an informed consent process. Microbiological tests were performed to confirm cholera cases by a stool culture growing *V*. *cholerae* O1 as the sole pathogen. Therefore we enrolled the patients who had watery diarrhea, and stool culture positive for *V*. *cholerae* and negative for other bacteria. We followed household contacts of cholera index patients in Dhaka, Bangladesh for 9 days following identification of an index case. Household contacts were defined as individuals who shared a cooking pot with the index case for three or more days prior to the cholera episode in the index case [6,7]. Within 24 hours of disease presentation of the index patient (day 2), household contacts were enrolled in the study. Contacts were questioned about their diarrheal symptoms on days 2–10 following presentation of the index case, and rectal swabs were obtained for *V*. *cholerae* culture from contacts regardless of diarrheal symptom.

Blood specimens were collected from the patients at day 2 during their hospital stay. Venous blood was also obtained from household contacts on days 2 and 7. Vibriocidal antibodies and IgA, IgG, and IgM antibodies to homologous serotype of *V*. *cholerae* O1 O-specific polysaccharide (OSP), lipopolysaccharide (LPS), as well as cholera toxin B subunit (CtxB) were assayed from plasma. Upon study enrollment, antigen-specific IgA, IgG, and IgM memory B cell levels were also measured from isolated peripheral blood mononuclear cells (PBMCs) of contacts and patients.

### Ethics statement

This study was approved by the Research Review Committee and Ethical Review Committee of the icddr,b, and the Institutional Review Board of the Massachusetts General Hospital. Informed written consent was obtained from all participants.

### Isolation of PBMCs and plasma

Heparinized blood was diluted in PBS; PBMCs and plasma were isolated by density gradient centrifugation using Ficoll-Isopaque (Pharmacia, Piscataway, NJ). Isolated plasma specimens were frozen at—20°C prior to use in immunologic analysis. We suspended PBMCs at a concentration of 1×10^7^ cells/ml in RPMI-complete medium (Gibco, Carlsbad, CA) containing 10% heat-inactivated fetal bovine serum (FBS; HyClone, Logan, UT). Re-suspended cells were used in the below described memory B cell assay.

### Plasma vibriocidal antibody assay

Vibriocidal antibody responses in plasma samples of patients and contacts were assessed as previously described using *V*. *cholerae* O1 Ogawa (X-25049) as the target organism [12]. We defined the vibriocidal titer as the reciprocal of the highest dilution resulting in >50% reduction of the optical density compared to that of control wells without plasma.

### OSP, LPS, and CtxB-specific IgA, IgG, and IgM antibody responses in plasma

Enzyme linked immunosorbant assays (ELISA) were performed to assess plasma IgA, IgG and IgM antibody response to OSP, LPS, and CtxB using standardized protocols [2,4,13]. Ninety six-well polystyrene plates (Nunc F, USA) were coated with *V*. *cholerae* O1 Ogawa OSP:BSA (1 μg/ml) dissolved in carbonate buffer (pH 9.6–9.8) for anti-OSP, O1 Ogawa LPS (2.5 μg/ml) dissolved in phosphate buffered saline (PBS) (pH 7.2–7.4) for anti-LPS, or 0.3 nmol of ganglioside GM1/ml followed by recombinant CtxB subunit (0.5 μg/ml) (gifts from A. M. Svennerholm, University of Gothenburg, Gothenburg, Sweden) for anti-CtxB responses. OSP was generated from *V*. *cholerae* O1 Ogawa strain PIC158 as previously described [2,14–16]. We added 100 μl of plasma (diluted 1:50 for OSP and LPS, and 1:200 for CtxB in 0.1% bovine serum albumin in phosphate-buffered saline–0.05% Tween) per well. Horseradish peroxidase-conjugated secondary antibodies to human IgG or IgA or IgM (Jackson Immunoresearch, West Grove, PA; 1:1000 dilution) were added to the wells, and plates were developed with ortho-phenylene diamine (Sigma, St. Louis, MO) in 0.1 M sodium citrate buffer (pH 4.5) and 0.012% hydrogen peroxide. ELISA plates were read kinetically at 450 nm for 5 min and the maximal rate of change in optical density was measured as milli-absorbance units per minute (mabs/min). ELISA units were normalized by calculating the ratio of the test sample to a standard of pooled convalescent-phase plasma from previously infected cholera patients considered as a positive control on each plate. We defined a plasma antibody responder as having a baseline ELISA unit value higher than that of the mean of the cohort plus three standard errors of the mean.

### Memory B cell culture and ELISPOT assay

Memory B cell assays were performed using recovered PBMCs on day 2 as previously described [4,7,17,18]. We placed 5 x 10^5^ PBMCs/well in cell culture plates (BD Biosciences, San Jose, CA) containing RPMI 1640 and 10% FBS. To stimulate antigen-independent proliferation and differentiation of memory B cells into ASCs (Antibody Secreting cells), a mixture of three B-cell mitogens containing 6 μg/ml CpG oligonucleotide (Operon, Huntsville, AL), a 1/100,000 dilution of crude pokeweed mitogen extract, and a 1/10,000 dilution of fixed *Staphylococcus aureus* Cowan (Sigma, St. Louis, MO) was added to all wells except those being used as negative controls, to which only media was added. Plates were incubated at 37°C in 5% CO_2_ for 5–6 days, after which the cells were harvested and washed.

For the memory B cell ELISPOT assay, we coated nitrocellulose-bottom plates (MSHAN-4550; Millipore, Bedford, MA) with OSP:BSA (10 μg/ml), LPS (25 μg/ml), or GM1 ganglioside (3 nmol/ml) followed by recombinant CtxB (2.5 μg/ml), 5 μg/ml affinity-purified goat anti-human immunoglobulin (Jackson Immunology Research, West Grove, PA) as a positive control, or 2.5 μg/ml keyhole limpet hemocyanin (KLH) (Pierce Biotechnology, Rockford, IL) as a negative control. After the plates were blocked with RPMI 1640 containing 10% FBS, 20% of the cells from each culture plate well were added for detection of total ASC and 80% were used for detection of antigen-specific ASC. Plates were incubated for 5 h at 37°C in 5% CO_2_, after that plates were washed, and alkaline phosphatase-conjugated goat anti-human IgG and horseradish peroxidase-conjugated goat anti-human IgA (Southern Biotech, Birmingham, AL) and horseradish peroxidase-conjugated mouse anti-human IgM (Hybridoma Reagent Laboratory, Baltimore, MD) were added at a dilution of 1:500. Following an overnight incubation at 4°C, plates were developed with 3-amino-9-ethyl carbazole (AEC) for IgA and IgM, or with BCIP (5-bromo-4-chloro-3-indolylphosphate)-nitroblue tetrazolium (NBT) for IgG.

We expressed ELISPOT counts as the percentage of antigen-specific memory B cells out of the total IgG, IgA, and IgM memory B cells. We used wells coated with KLH and un-stimulated samples as negative controls. In this study, we defined appropriate stimulation of PBMC as a 3-fold increase in the number of total immunoglobulin memory cells after stimulation compared to un-stimulated cells. We counted the number of memory B cell ASC per well independently by two reviewers in a sample-blinded fashion using a stereomicroscope, and averaged the counts. We excluded data from analysis for any of the following reasons: if, (i) the total immunoglobulin samples for each patient sample did not have appropriate stimulation, (ii) the study subject specimens had four or more antigen-specific ASC spots in the same sample prior to stimulation to exclude acute infection and exposure to *V*. *cholerae* O1 antigens and other related pathogens, or (iii) patient samples had four or more ASC spots to the negative control antigen KLH, as previously described [6,7].

### Definition of infected and un-infected contacts and exclusion criteria

Household contacts were defined as infected or un-infected on the basis of the culture results of 9 days of rectal swabs and 4 fold or greater increase of vibriocidal titer between day 2 and day 7, as previously defined [7]. Household contacts who had *V*. *cholerae* O1 positive in the rectal swab culture at least once during the day 2 through day 10 follow up period, and/or 4-fold or greater increase of vibriocidal titer at day 7 compared to day 2 were classified as infected contacts. Our previous studies [4,17,19] showed that *V*. *cholerae* infected patients developed higher vibriocidal antibody titer at day 7 compared to day 2, and we defined them as a responder if they had 4 fold or greater increase of vibriocidal titer at day 7. Therefore, contacts who had 4 fold or greater increase of vibriocidal titer can be possibly considered as infected. Contacts with no positive rectal swabs and no 4-fold increase of vibriocidal titer between day 2 and day 7, and no symptoms of diarrhea during the day 2–10 follow up were defined as un-infected contacts. Contacts who did not follow either criteria were defined as unclassified and excluded from the immunologic analysis. Contacts were also excluded from analysis if they had diarrhea in the week before enrollment, had *V*. *cholerae* infection with a different sero-group or serotype than the corresponding index case, or did not complete the follow up. Index patients who had symptoms of diarrhea for more than 24 hours prior to presentation were also excluded from analysis. Number of infected contacts from rectal swab positive specimen is 54; increased vibriocidal titer is 37 and from both 25.

### Statistical analysis

We assessed the differences in the magnitude of responses using Mann-Whitney U tests, and used χ^2^ tests to assess relative risk ratios with confidence intervals. All reported P values were two-tailed, with a cutoff of P < 0.05 considered a threshold for statistical significance. Data analysis and figure preparation were performed using Graphpad Prism 5.0 (GraphPad Software, Inc., La Jolla, CA) and SPSS 14 (SPSS Inc., Chicago, IL).

## Results

### Study population

**Fig 1:**
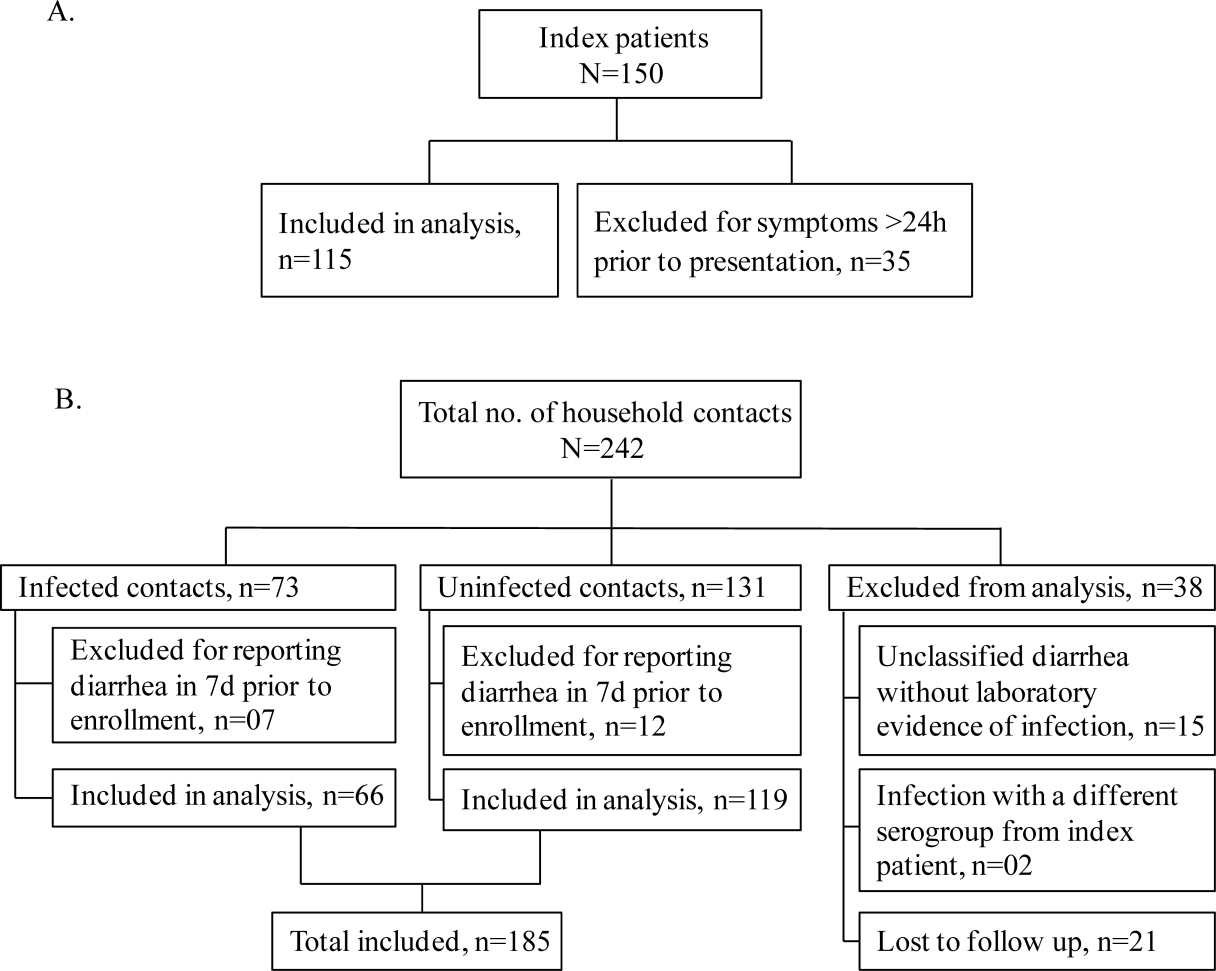
Enrollment and classification of study participants (A-cholera index patients, and B-household contacts of index patients). Infected and un-infected contacts who had no diarrhea in the week before presentation were included in the immunologic analysis. Index patients, who had symptoms of diarrhea equal or less than 24 hours before enrollment, were also included in the analysis.

A total of 242 household contacts of 150 index patients with cholera were enrolled in this study between February-2012 and December-2015. The age range of index patients and their household contacts was 2–60 years. The average number (median) of household contacts per index patients was 1 (range 1–5). All patients were infected with *V*. *cholerae* O1 Ogawa. Study participants were categorized into different groups as shown in Fig 1. Of the 150 enrolled patients, 115 had symptoms for 24 hours or less prior to presentation at the icddr,b hospital and were included in the immunologic analysis; 35 were excluded for having symptoms for more than 24 hours prior to presentation. Of the 242 contacts enrolled, 73 were categorized as infected contacts, 131 were categorized as uninfected, and 38 were excluded from analysis for unclassified diarrhea without laboratory evidence of infection (N = 15), for infection with a different serogroup or serotype from index patient (N = 2), or for loss of follow-up (N = 21). For reporting diarrhea in the week prior to enrollment, 7 were excluded from infected contacts and 12 were excluded from uninfected contacts. As a result, in total 185 were included in the analysis (Fig 1). Demographic and clinical characteristics of study participants are shown in Table 1.

**Table 1:** Demographic characteristics of study participants.

Characteristics		Value for group		
		Patients (115)	Infected Contacts (66)	Un-infected Contacts (119)
Age (yrs), median (range)*		25 (2–51)	26 (3–60)	30 (4–56)
No. (%) female		35 (30.4)	37 (56.1)	74 (62.2)
No. (%) with ABO blood group	O	48 (41.7)	18 (27.3)	27 (22.7)
	A	26 (22.6)	18 (27.3)	43 (36.1)
	B	31 (27.0)	26 (39.4)	39 (32.8)
	AB	10 (8.7)	4 (6.1)	10 (8.4)

* No difference of age between the groups has been found.

### Vibriocidal responses

**Fig 2:**
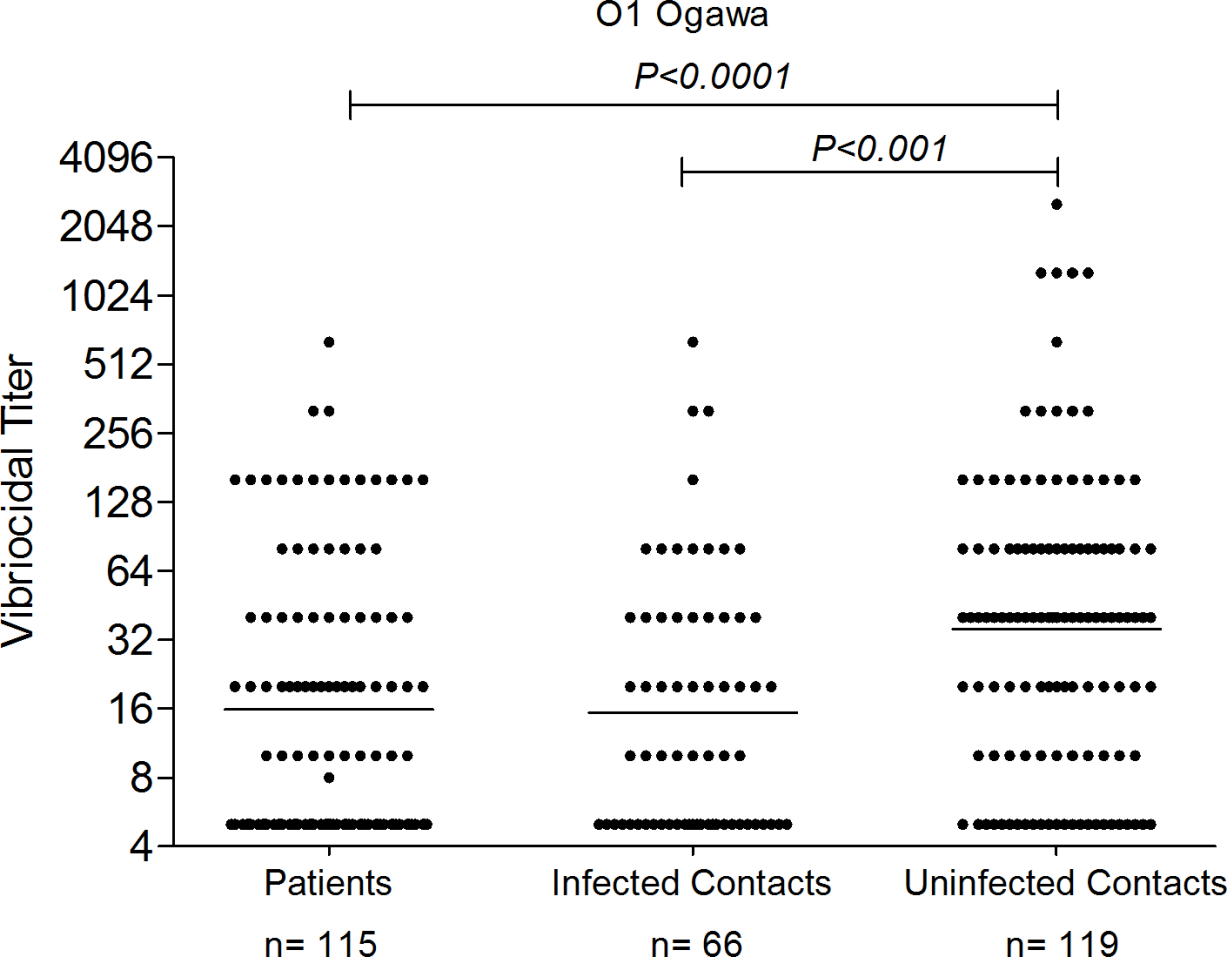
Plasma vibriocidal antibody titers upon enrollment. Bars represent geometric mean responses. *P* values for statistical significant differences between groups determined by Mann-Whitney U test.

The baseline geometric mean (GM) reciprocal vibriocidal titer (GMT) in un-infected contacts was 36 (95% confidence interval [CI]: 27 to 47); in infected contacts it was 15 (95% confidence interval [CI]: 11 to 21), and in patients it was 16 (95% confidence interval [CI]: 12 to 20). Un-infected contacts demonstrated the highest baseline vibriocidal titers, with these values being significantly higher than those of infected contacts (p<0.001) and patients (p<0.0001) (Fig 2).

### OSP, LPS, and CtxB-specific antibody responses

**Fig 3:**
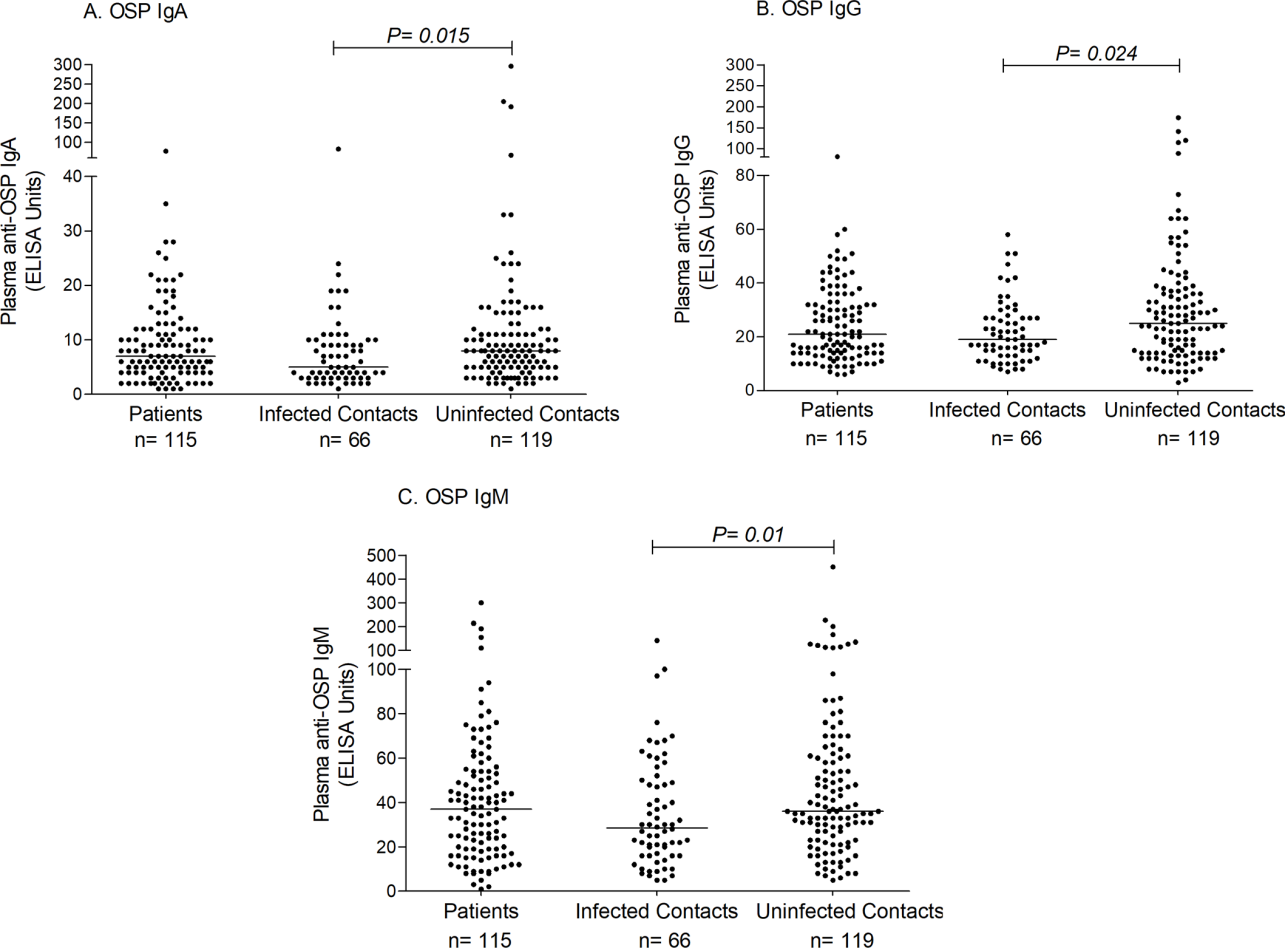
**Plasma OSP-specific IgA, IgG and IgM antibody values (A, B and C, respectively) upon enrollment (day 2).** Bars represent median responses. *P* values for statistical significant differences between groups determined by Mann-Whitney U test.

We assessed baseline plasma OSP, LPS, and CtxB-specific IgA, IgG, and IgM antibody responses in household contacts and index patients on the day of enrollment (day 2). Un-infected contacts had the highest baseline plasma OSP-specific IgA, IgG and IgM antibody responses, and these values were significantly higher than that of infected contacts (p = 0.015, p = 0.024 and p = 0.01, respectively; Fig 3). Having a baseline OSP-specific IgG value higher than the mean of the cohort plus three standard errors of the mean was associated with a risk ratio of becoming infected of 0.62 (95% confidence interval [CI], 0.403 to 0.964; Table 2).

**Table 2:** Risk of infection among contacts with plasma OSP-specific antibody values*.

**All contacts**		
**Responder for plasma antibody titer**	**Risk ratio**	**95% CI**
OSP IgA	0.65	0.358 to 1.193
OSP IgG	0.62	0.403 to 0.964
OSP IgM	0.77	0.498 to 1.178
**Contacts with d2 vibriocidal titer <160**		
**Responder for plasma antibody titer**	**Risk ratio**	**95% CI**
OSP IgA	0.71	0.442 to 1.140
OSP IgG	0.71	0.453 to 1.098
OSP IgM	0.85	0.555 to 1.306

*Comparing contacts with OSP-specific antibody values more than the cohort mean plus three times of the standard error of the mean, with all others in the cohort.

Baseline plasma LPS-specific IgA, IgG and IgM antibody responses mirrored those seen for OSP, although the differences in values in infected versus un-infected household contacts did not reach significance (S1 Fig). When considering baseline plasma CtxB-specific responses, only baseline IgA values were higher in un-infected contacts versus infected contacts (p = 0.03; S2 Fig).

### Antigen-specific memory B cell responses

**Fig 4:**
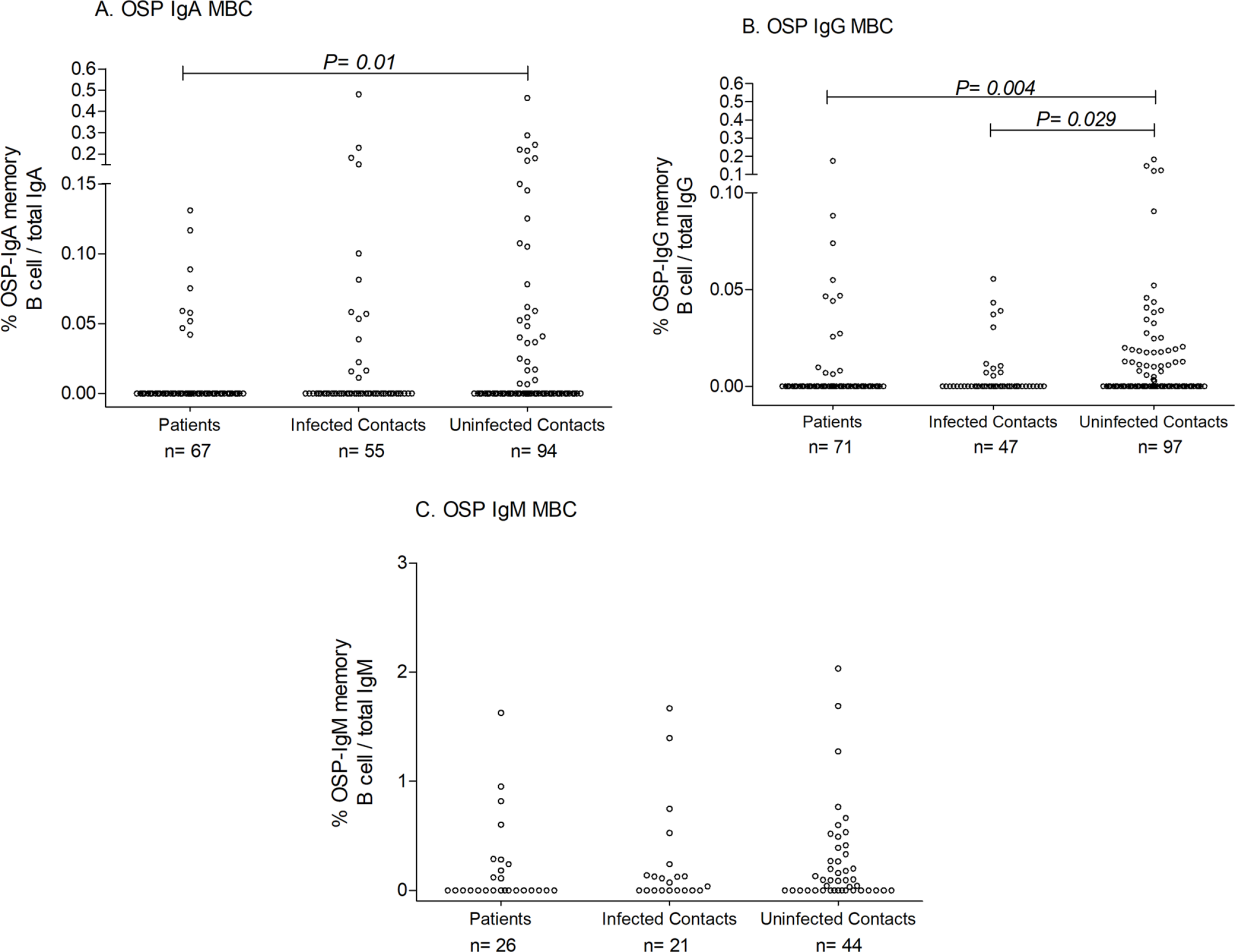
**OSP-specific IgA (A), IgG (B) and IgM (C) memory B cell responses upon enrollment (day 2).** *P* values for statistical significant differences between groups determined by Mann-Whitney U test.

**Fig 5:**
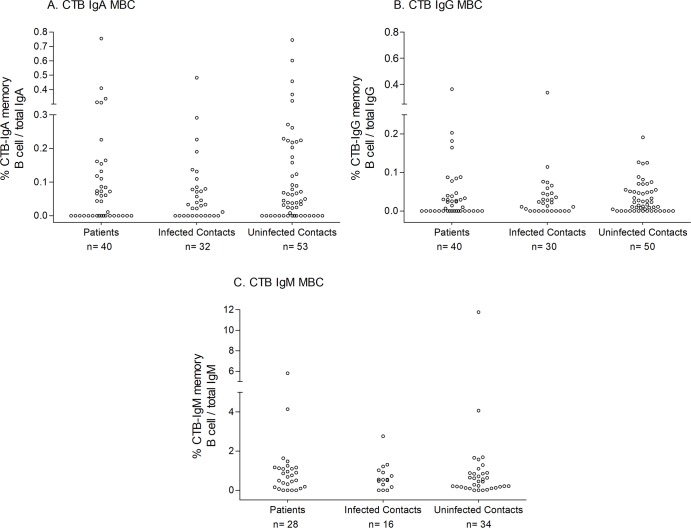
CTB-specific IgA, IgG and IgM memory B cell responses upon enrollment (day 2) (A, B and C, respectively).

We prioritized assessing OSP and LPS-specific memory B cell responses over CtxB, and determining IgG and IgA memory B cell responses over IgM when PBMCs were limiting (Fig 4 and S3 Fig). Baseline OSP and LPS-specific IgG memory B cell responses were higher in un-infected versus infected household contacts (p = 0.029 and p = 0.039, respectively; Fig 4B and S3B Fig). We did not observe any significant differences in OSP and LPS-specific IgA and IgM memory B cell responses between un-infected and infected household contacts, although baseline OSP IgA and LPS IgM memory B cell values were higher in un-infected contacts compared to cholera index patients (OSP IgA, p = 0.01; LPS IgM, p = 0.007; Fig 4A and S3C Fig). The presence of detectable OSP-specific or LPS-specific IgG memory B cell values at baseline was associated with a significant decrease in the risk of infection in contacts (Table 3; OSP: risk ratio 0.56; 95% confidence interval [CI] 0.311 to 0.998; LPS: risk ratio 0.59; 95% confidence interval [CI] 0.367 to 0.934). Vibriocidal titer has been considered as the surrogate marker of protection against cholera, and therefore, when considering only contacts with baseline vibriocidal values of 1:80 or lower, the presence of LPS IgG memory B cells was also significantly associated with a decreased risk of infection in contacts, and the risk reduction when OSP IgG memory B cells were present approached significance (OSP: risk ratio 0.62; 95% confidence interval [CI] 0.350 to 1.100; LPS: risk ratio 0.62; 95% confidence interval [CI] 0.384 to 0.987). We found no association of memory B cells of any isotype targeting CtxB and risk of infection (Fig 5).

**Table 3:** Risk of infection for contacts with detectable LPS- and OSP-specific memory B cell (MBC) responses relative to those without detectable MBC.

MBC detected		Risk ratio	95% CI
LPS IgA	All contacts	1.28	0.738 to 2.226
	Contacts with d2 vibriocidal titer <160	1.22	0.702 to 2.137
LPS IgG	All contacts	0.59	0.367 to 0.934
	Contacts with d2 vibriocidal titer <160	0.62	0.384 to 0.987
OSP IgA	All contacts	0.84	0.514 to 1.378
	Contacts with d2 vibriocidal titer <160	0.90	0.557 to 1.455
OSP IgG	All contacts	0.56	0.311 to 0.998
	Contacts with d2 vibriocidal titer <160	0.62	0.350 to 1.100

## Discussion

In this study, we found that uninfected contacts developed higher plasma antibody and memory B cells responses to OSP than infected contacts. These growing bodies of evidence suggest that the O-specific polysaccharide (OSP) component of *V*. *cholerae* is the target immunogen involved in mediating protection against cholera [2–7]. Previous infection with *V*. *cholerae* O1 provides no protection against O139, and vice versa [12,20,21]. This is despite the fact that the organisms are largely identical at the genomic level and express very similar cholera toxins. The main difference between *V*. *cholerae* O1 and O139 is in the genes encoding OSP and presence or absence of capsule comprised of OSP [15,22–26].

Historically, the best marker of protection against cholera has been considered the vibriocidal assay. In the current study, we also found that uninfected contacts had higher vibriocidal titers than patients and infected contacts. The vibriocidal assay assesses cell-independent complement-mediated bacteriolysis in an in vitro assay. Vibriocidal antibodies largely target *V*. *cholerae* LPS; specifically the O-specific polysaccharide component of LPS [2]. Since mucosal IgA does not bind complement; and since there is minimal if any component of the terminal attack complex in the intestinal lumen when intestinal epithelium is intact, it is thought that the vibriocidal assay is largely a surrogate marker for as yet poorly defined mucosal immune responses, possibly targeting LPS/OSP. We have previously shown that baseline plasma IgA responses, but not IgG responses, against LPS and CtxB correlate with protection against cholera in an analysis of household contacts of cholera index patients [6]. In our current study we have found that uninfected contacts had higher LPS IgA response than patients (S1 Fig). We have also shown that fecal IgA responses targeting LPS, but not CtxB, also correlate with protection against cholera [6]. In this current study, we extend this observation and now show that IgA, IgM, and IgG immune responses targeting OSP also correlate with protection against cholera in household contacts of cholera index patients.

Despite such associations, we have previously shown that anti-*V*. *cholerae* antibody responses in plasma are relatively short lived [17,19]. Vibriocidal and anti-LPS and anti-OSP immune responses peak within 7 days of infection and fall back toward baseline within 6 to 12 months [2,4,17,19]. This is despite the fact that previous infection with cholera induces protection against symptomatic disease of at least 3 to 10 years [9–11]. Memory B cells against *V*. *cholerae* antigens may be correlate with long term protection. Because, patients recovering from cholera also develop prominent memory B cell responses against LPS, OSP, and CtxB [3,4,17,19]. These responses are detectable for much longer periods than short-lived plasma effector antibody responses [17,19], and we assume they correlate with either the presence of memory B cells or long-lived plasma cells in lamina proprial intestinal tissue, and that these cells allow a rapid anamnestic response upon re-exposure and protection from subsequent disease. We have previously shown that baseline memory B cell responses targeting *V*. *cholerae* LPS, but not CtxB, correlate with protection against cholera among household contacts of cholera index patients in Dhaka, Bangladesh [7]. We have also previously shown that the antibody response to *V*. *cholerae* LPS primarily targets the OSP antigen [8]. We were therefore interested in also assessing whether memory B cell responses targeting the OSP component of LPS also correlate with protection against cholera since OSP mediates the sero-specificity of LPS. In this current study, we found that this is indeed the case. OSP memory B cell IgG responses correlated with protection against infection, while CtxB memory B cell responses did not. We also note higher baseline memory B cell IgA and IgM OSP values and protection among household contacts compared to infected contacts, although these differences did not reach statistical significance in our current study. Whether this represents a real difference regarding memory B cell isotype or is a reflection of our cohort sample size is currently unclear.

In a hyperendemic cholera area such as Dhaka, Bangladesh, previous and repetitive exposure to *V*. *cholerae* might be a potent inducer of IgG memory B cell responses against OSP, a T cell independent antigen. Immune responses of the IgM and IgA isotype among memory B cells are of lower absolute value and may be harder to distinguish from background reactivity. In either case, it is unlikely that systemically circulating memory B cells of the IgG isotype are direct mediators of long-term protection against cholera. *V*. *cholerae* is a noninvasive mucosal pathogen, and presumably the memory B cell responses that we detected in this study correlate with immune responses that are directly at the mucosal surface. Our results, however, suggest that long-lived immune responses that target *V*. *cholerae* OSP are associated with protection against cholera. It is uncertain that such immune responses are induced by currently available cholera vaccines.

We have previously shown that memory B cell responses induced following vaccination with a killed oral cholera vaccine are much more blunted than in age-matched individuals recovering from wild-type cholera in Bangladesh [17,27]. Memory B cell responses in young children under the age of 5 targeting *V*. *cholerae* LPS/OSP were also particularly blunted [27,28]. These observations may suggest a reason for the relatively poor and short-term protection afforded to young children by vaccination with this vaccine. Whether other oral cholera vaccines induce memory B cell or long-lived plasma cell responses, and the duration of protection afforded by such vaccination are currently unclear and require investigation.

Our study has a number of limitations. We did not assess for the presence of either long-lived plasma cells or memory B cells directly in intestinal tissue due to practical constraints, although such immune responses may be the best predictor and actual mediators of long-lived protection against cholera. Also, our study only investigated patients and contacts with *V*. *cholerae* Ogawa infection, since that was the predominant circulating serotype in Dhaka at the time of the analysis. In addition, our study does not address at a mechanistic level how antibodies targeting *V*. *cholerae* OSP might actually mediate protection against cholera. Despite these limitations, we found that immune responses targeting *V*. *cholerae* OSP, including both plasma anti-OSP responses and long-lived memory responses, correlate with protection against cholera in humans in Bangladesh. This observation further supports the hypothesis that immune responses that target *V*. *cholerae* OSP is a prime mediator of protection against cholera, and suggests that future work should focus on more detailed analysis of mucosal immune responses targeting OSP, as well as evaluation of potential mechanisms of functional protection against cholera afforded by immune responses targeting OSP.

## Supporting information

S1 Fig **Plasma LPS-specific IgA, IgG and IgM antibody responses (A, B and C, respectively) upon enrollment (day 2).** Bars represent median responses. *P* values for statistical significant differences between groups determined by Mann-Whitney U test.
(PDF)

S2 Fig **Plasma CTB-specific IgA and IgG antibody responses (A and B, respectively) upon enrollment (day 2).** Bars represent median responses. *P* values for statistical significant differences between groups determined by Mann-Whitney U test.
(PDF)

S3 Fig **LPS-specific IgA, IgG and IgM memory B cell responses upon enrollment (day 2) (A, B and C, respectively).** *P* values for statistical significant differences between groups determined by Mann-Whitney U test.
(PDF)
