# Correction: Plasma and memory B cell responses targeting O-specific polysaccharide (OSP) are associated with protection against *Vibrio cholerae* O1 infection among household contacts of cholera patients in Bangladesh

**DOI:** 10.1371/journal.pntd.0009616

**Published:** 2021-07-19

**Authors:** Amena Aktar, M. Arifur Rahman, Sadia Afrin, Aklima Akter, Taher Uddin, Tahirah Yasmin, Md. Israk Nur Sami, Pinki Dash, Sultana Rownok Jahan, Fahima Chowdhury, Ashraful I. Khan, Regina C. LaRocque, Richelle C. Charles, Taufiqur Rahman Bhuiyan, Anjali Mandlik, Meagan Kelly, Pavol Kováč, Peng Xu, Stephen B. Calderwood, Jason B. Harris, Firdausi Qadri, Edward T. Ryan

The de-identified, individual-level data set underlying the charts and statistics is not included in the published article and its Supporting Information files. The authors have provided the data as Supporting Information file S1 File.

## Supporting information

S1 File (XLSX)
