# Supplementary figures and images for: Plasma and memory B cell responses targeting O-specific polysaccharide (OSP) are associated with protection against Vibrio cholerae O1 infection among household contacts of cholera patients in Bangladesh
Source: PLoS Negl Trop Dis. 2018 Apr 23;12(4):e0006399. doi: 10.1371/journal.pntd.0006399 (PMC5912711; doi:10.1371/journal.pntd.0006399)

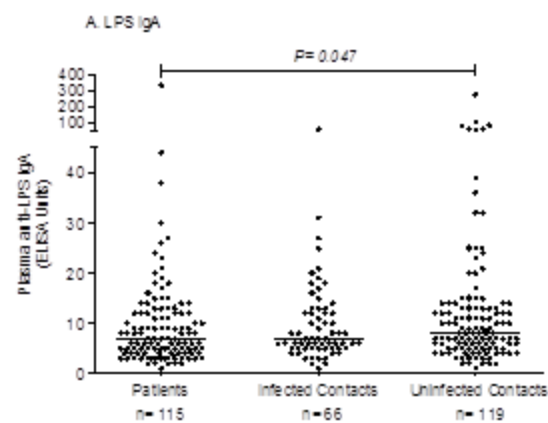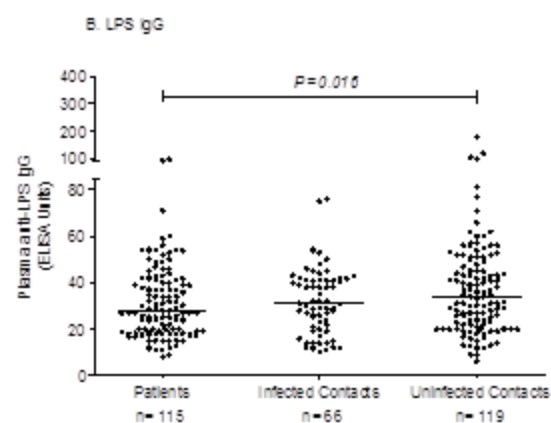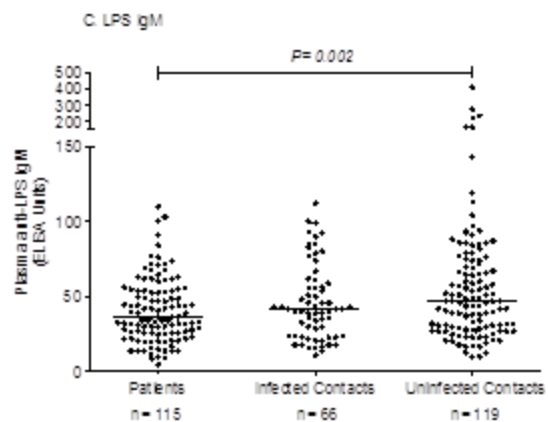

Supplement: S1 Fig — Plasma LPS-specific IgA, IgG and IgM antibody responses (A, B and C, respectively) upon enrollment (day 2). Bars represent median responses. P values for statistical significant differences between groups determined by Mann-Whitney U test. (PDF) [file pntd.0006399.s001.pdf]

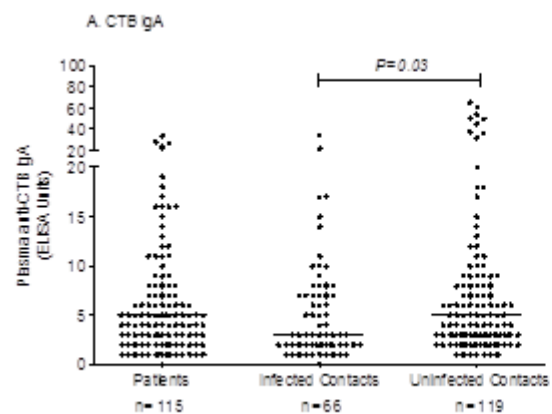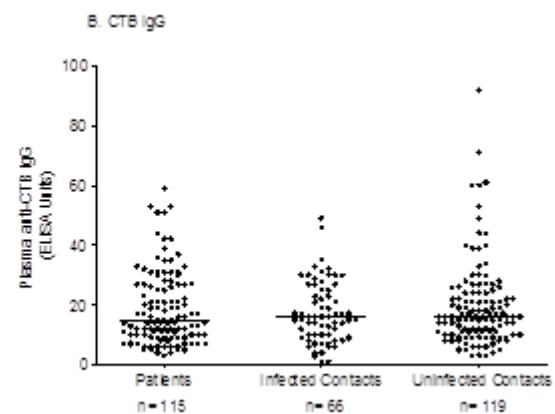

Supplement: S2 Fig — Plasma CTB-specific IgA and IgG antibody responses (A and B, respectively) upon enrollment (day 2). Bars represent median responses. P values for statistical significant differences between groups determined by Mann-Whitney U test. (PDF) [file pntd.0006399.s002.pdf]

A. LPS IgA MBC

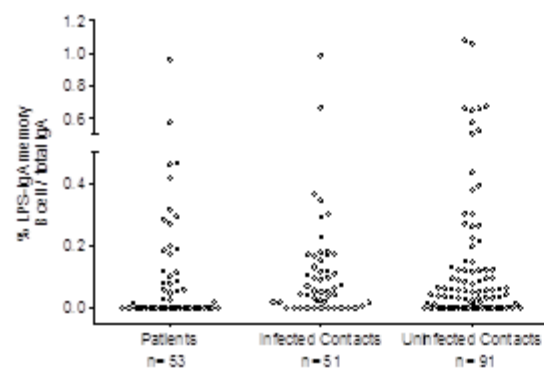

B. LPS IgG MBC

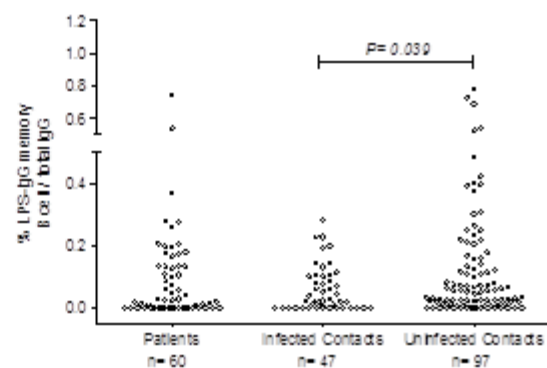

C. LPS IgM MBC

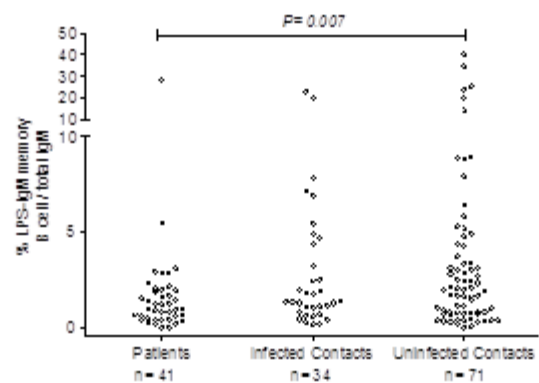

Supplement: S3 Fig — LPS-specific IgA, IgG and IgM memory B cell responses upon enrollment (day 2) (A, B and C, respectively). P values for statistical significant differences between groups determined by Mann-Whitney U test. (PDF) [file pntd.0006399.s003.pdf]
